# A Simple Radiological Technique for Demonstration of Incorrect Positioning of a Foley Catheter with Balloon Inflated in the Urethra of a Male Spinal Cord Injury Patient

**DOI:** 10.1100/tsw.2006.381

**Published:** 2006-06-20

**Authors:** Subramanian Vaidyanathan, Peter L. Hughes, Bakul M. Soni

**Affiliations:** ^1^ Regional Spinal Injuries Centre, Southport, Merseyside, UK; ^2^ District General Hospital, Southport, Merseyside, UK

**Keywords:** spinal cord injury, Foley catheter, urethra

## Abstract

In a male patient with cervical spinal cord injury, the urinary bladder may go into spasm when a urethral catheter is removed and a new Foley catheter is inserted. Before the balloon is inflated, the spastic bladder may push the Foley catheter out or the catheter may slip out of a small-capacity bladder. An inexperienced health professional may inflate the balloon of a Foley catheter in the urethra without realizing that the balloon segment of the catheter is lying in the urethra instead of the urinary bladder. When a Foley balloon is inflated in the urethra, a tetraplegic patient is likely to develop autonomic dysreflexia. This is a medical emergency and requires urgent treatment. Before the incorrectly placed Foley catheter is removed, it is important to document whether the balloon has been inflated in the urinary bladder or not. The clinician should first use the always available tools of observation and palpation at the bedside without delays of transportation. A misplaced balloon will often be evident by a long catheter sign, indicating excessive catheter remaining outside the patient. Radiological diagnosis is not frequently required and, when needed, should employ the technique most readily available, which might be a body and pelvic CT without intravenous contrast. An alternative radiological technique to demonstrate the position of the balloon of the Foley catheter is described. Three milliliters of nonionic X-ray contrast medium, Ioversol (OPTIRAY 300), is injected through the side channel of the Foley catheter, which is used for inflating the balloon. Then, with a catheter-tip syringe, 30 ml of sterile Ioversol is injected through the main lumen of the Foley catheter. Immediately thereafter, an X-ray of the pelvis (including perineum) is taken. By this technique, both the urinary bladder and balloon of the Foley catheter are visualized by the X-ray contrast medium. When a Foley catheter has been inserted correctly, the balloon of the Foley catheter should be located within the urinary bladder, but when the Foley catheter is misplaced with the balloon inflated in the urethra, a round opaque shadow of the Foley balloon is seen separately below the urinary bladder. This radiological study takes only a few minutes to perform, can be carried out bedside with a mobile X-ray machine, and does not require special expertise or preparations, unlike transrectal ultrasonography. When a Foley balloon is inflated in the urethra, abdominal ultrasonography will show an absence of the Foley balloon within the bladder. The technique described above aids in positive demonstration of a Foley balloon lying outside the urinary bladder. Such documentation proves valuable in planning future treatment, education of health professionals, and settlement of malpractice claims.

## INTRODUCTION

Very rarely, the tip of a Foley catheter may be misplaced inside the ureter, thus causing hydroureteronephrosis[1]. When the balloon of a Foley catheter is inflated within a vesical diverticulum, appearances of extraluminal location of the balloon in computerized tomography (CT) of the pelvis may simulate sealed bladder perforation[2]. Persons with cervical spinal cord injury are often managed by a long-term, indwelling, urethral Foley catheter. These patients are at risk of incorrect positioning of the Foley catheter, albeit such accidents happen only rarely.

## INCORRECT POSITIONING OF A FOLEY CATHETER WITH THE BALLOON INFLATED IN THE URETHRA: HOW DOES IT HAPPEN?

When an indwelling catheter is removed and a new Foley catheter is inserted in a male tetraplegic patient, the bladder may go in to spasm. Before the balloon is inflated, the spastic bladder may push the Foley catheter out or the catheter may slip out of a small-capacity bladder. An inexperienced health professional may then inflate the balloon of the Foley catheter without realizing that the catheter has partly slipped out and the balloon segment of the catheter is presently located in the urethra instead of lying within the urinary bladder. Very rarely, when a Foley catheter is inserted per urethra, urine may start trickling out as the tip of catheter enters the bladder neck; a novice may then inflate the balloon hurriedly while the balloon segment is still located in proximal urethra.

## INFLATING THE FOLEY BALLOON IN THE URETHRA OF A TETRAPLEGIC PATIENT: CLINICAL SYMPTOMS — AUTONOMIC DYSREFLEXIA: A MEDICAL EMERGENCY

When the balloon of a Foley catheter is inflated in the urethra, a patient with complete paralysis will not experience pain, but an astute patient will notice that an unusually long segment of the Foley catheter is lying outside the penis. Cervical spinal cord injury patients are likely to develop increased spasms and manifest features of autonomic dysreflexia such as sweating and headache. This is a medical emergency and the catheter needs repositioning urgently, otherwise persistent autonomic dysreflexia can lead to dire consequences such as intracranial bleeding and fatality. Before the balloon of the Foley catheter is deflated, it is important to document the location of the catheter as to whether the balloon had been inflated in the bladder or not. This assumes great importance, especially for medico-legal reasons.

The clinician should first use the always available tools of observation and palpation at the bedside without delays of transportation. A misplaced balloon will often be evident by a *long catheter sign*, indicating an excessive length of catheter remaining outside the patient. Blood may be seen at the external urethral meatus, which is an indication of urethral trauma. The Foley balloon, inflated in the deep bulbous urethra, can be felt on palpation of the perineum or combined rectal examination. Further confirmation of the problem can be achieved by attempted catheter irrigation. If a catheter is inserted inside the urinary bladder, there will be free return of the irrigation fluid, indicating that the tip of the Foley catheter is in a free fluid filled space. In contrast, a catheter placed in the urethra will accept fluid inserted, which then may travel into the bladder, but will not have free fluid return on aspiration, as the tip of the Foley is not located in a free fluid filled space. These clinical signs are the primary tools employed in the diagnosis of a misplaced Foley catheter. Radiological diagnosis is not frequently required, but when needed, should employ the technique most readily available, which might be an abdomen and pelvis CT without intravenous contrast, as such service is often available in most emergency departments and can be rapidly obtained by the radiology technician on call. In spinal injury centers where facilities for CT, especially during off hours, may not be available, the following technique may prove useful. However, we should point out that elaborate radiological diagnosis is not a substitute for prompt clinical assessment and treatment.

## DESCRIPTION OF RADIOLOGICAL TECHNIQUE TO SHOW THE LOCATION OF THE FOLEY BALLOON

We describe a simple radiological technique to document the position of the balloon of a Foley catheter. This technique can be performed bedside with a mobile X-ray machine; it does not require any special preparations and takes only a few minutes to carry out. The patient is placed supine and preparations are made to take an X-ray of the pelvis (including the perineum). Three milliliters of nonionic X-ray contrast medium, Ioversol (OPTIRAY 300), is injected through the side channel of the Foley catheter, which is used for inflating the balloon. Then with a catheter-tip syringe, 30 ml of sterile Ioversol is injected through the main lumen of the Foley catheter. Immediately thereafter, radiological exposure is made. By this technique, the urinary bladder as well as the balloon of the Foley catheter are visualized. Under normal circumstances, the balloon of the Foley catheter is located within the urinary bladder, but when the balloon of the Foley catheter is inflated in the urethra, a round opaque shadow of the Foley balloon is seen separately below the urinary bladder (Fig. 1), thus clinching the diagnosis of incorrect positioning of the Foley catheter with the balloon inflated in the urethra.

Immediately after the radiological study, the side channel is aspirated and the Foley balloon is deflated. Prompt removal of contrast medium from the Foley balloon after radiological documentation of the location of the balloon, vis-à-vis urinary bladder, will preclude any rare possibility of clogging the balloon channel by the contrast medium. The aim of treatment is to establish bladder drainage promptly and urethral continuity if possible. A well-lubricated *coude* Foley may accomplish this rapidly at the bedside with swift improvement in symptoms of autonomic hyper-reflexia, when present. We prefer using flexible cystoscopy to insert a 0.032” guide wire into the urinary bladder; then a 16 Fr. Foley catheter is threaded over the guide wire. Use of flexible cystoscope will ensure accurate positioning of the Foley catheter in the bladder and prevent the Foley catheter from getting curled up in the dilated urethra[3].

Fluoroscopy or flexible cystoscopy entails services that may not be available immediately. At times when urethral catheterization is not easily achieved, a percutaneous cystostomy for bladder drainage may be performed rapidly with or without ultrasound guidance.

## ADVANTAGES OF THIS RADIOLOGICAL TECHNIQUE OVER ABDOMINAL ULTRASONOGRAPHY AND TRANSRECTAL ULTRASONOGRAPHY

When the balloon is inflated in the urethra, abdominal ultrasonography will show that the balloon of the Foley catheter is not visible inside the urinary bladder. However, it may be difficult to visualize the urinary bladder by sonography if the bladder is empty or if the patient is having a severe spasm of abdominal muscles. Distension of the urinary bladder in order to facilitate sonography may worsen the autonomic dysreflexia. Further, what is required is a *positive* demonstration of the precise location of the balloon of the Foley catheter. Is the balloon located in the bladder or in the urethra?

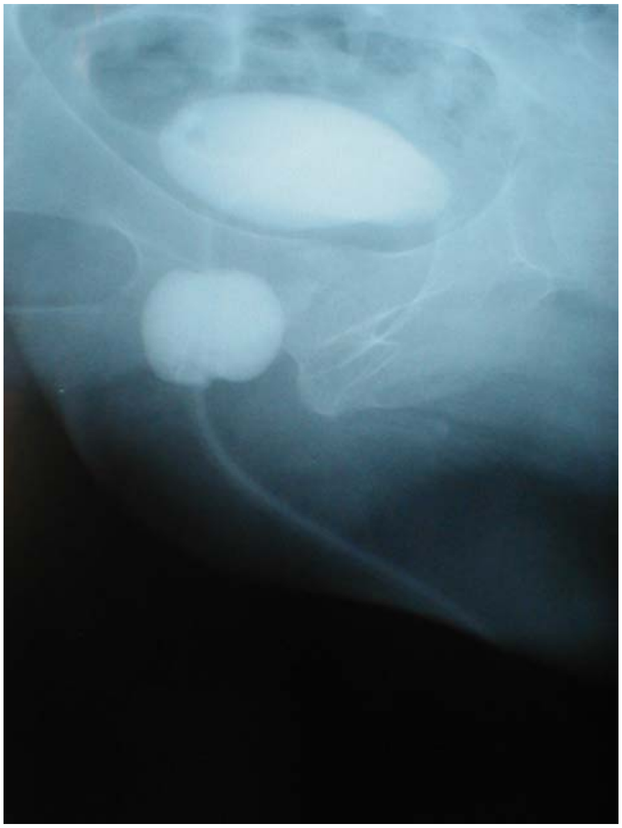

**FIGURE 1.** X-ray of pelvis is taken after injecting 30 ml of sterile nonionic X-ray contrast medium, Ioversol, through the main lumen of the Foley catheter (for visualizing the urinary bladder) and 3 ml of Ioversol through the side channel. Injecting contrast medium through the side channel enables visualization of the balloon of the Foley catheter. The round opaque shadow, which is located below the urinary bladder, represents the balloon of the Foley catheter. The Foley catheter can be seen as it traverses the distal urethra because the catheter is delineated by the radio-opaque contrast medium within its lumen. This radiological study takes less than 5 min to perform and clinches the diagnosis that the balloon of the Foley catheter is located in the urethra, an invaluable aid in planning future treatment and settling malpractice claims.

Transrectal ultrasonography[4] will show the position of the Foley balloon in relation to the bladder neck, but facilities for transrectal ultrasonography may not exist in spinal injury units or spinal cord physicians may not have expertise in performing transrectal ultrasonography. In some spinal cord injury patients, the rectum may be loaded with feces and transrectal ultrasongraphy becomes a very embarrassing procedure for patients and health professionals alike. Manual evacuation of feces will have to be performed in these patients before a rectal probe can be inserted and one is able to achieve adequate sonographic visualization of the bladder neck and Foley catheter. Further, anorectal procedures that involve the stretching of the anal sphincter may cause significant blood pressure elevation in susceptible patients. Topical lidocaine may prove inadequate to limit or prevent autonomic dysreflexia significantly[5].

## POTENTIAL SHORTCOMINGS OF THIS RADIOLOGICAL TECHNIQUE

The technique described herein will identify the location of a Foley catheter. However, it requires radiology services including a radiologist to administer contrast. In our spinal unit, a spinal cord physician who is trained in lower urinary tract radiology is able to carry out this procedure at the patient's bedside. Another potential problem that may occur is worsening of urethral trauma caused by further inflation of the Foley balloon. Therefore, we are cautious and inject no more than 3 ml of contrast through the balloon channel. Worsening of urethral trauma, when it occurs, may aggravate symptoms of autonomic hyperreflexia in turn. We administer nifedipine 10 mg sublingually should a patient develop symptoms of autonomic dysreflexia. If radiology services and personnel are not immediately available, patients suffering from a traumatic and misplaced Foley catheter may experience delay in diagnosis and treatment, which can be life threatening particularly to those patients with autonomic hyperreflexia. When radiological demonstration is needed, the most quickly available definitive test should be considered, which might be the CT of the abdomen and pelvis rather than the presented technique. However, in spinal injury units where facilities for CT, especially during off hours, may not be readily available, the technique described above is likely to prove useful.

## CLINICAL HISTORY AND MANAGEMENT OF AN ILLUSTRATIVE CASE

The X-ray shown in Fig. 1 was taken of a 36-year-old male patient with C-4 tetraplegia, in whom a district nurse had changed an indwelling Foley catheter. Following routine change of a urethral catheter, this patient developed profuse sweating and headache. Therefore, he was rushed to a spinal injuries center. Radiological study, performed within a few minutes of arrival in the spinal unit, demonstrated incorrect positioning of the Foley catheter with the balloon inflated in the urethra. In this patient, a new catheter was inserted with a flexible cystoscope. Following satisfactory positioning of Foley catheter, features of autonomic dysreflexia subsided. Documentation of incorrect positioning of the Foley catheter proves valuable in the education of health professionals and settlement of malpractice lawsuits.

## ACKNOWLEDGMENT

The authors are grateful to Dr. Vikram Dogra, Editor of *TSW Urology* for guidance and help in publication of this manuscript.

## References

1. George, J. and Tharion, G. (2005) Transient hydroureteronephrosis caused by a Foley's catheter tip in the right ureter. *TheScientificWorldJOURNAL* **5,** 367–369.
2. Abadi, S., Brook, O.R., Solomonov, E., and Fischer, D. (2006) Misleading positioning of a Foley catheter balloon. *Br. J. Radiol*. **79(938),** 175–176.
3. Vaidyanathan, S., Krishnan, K.R., and Soni, B.M. (1996) Endoscopic management of urethral trauma in male spinal cord injury patients. *Spinal Cord* **34(11),** 651–656.
4. St. Luce, S., Ninan, A.C., Hall, J.A., Kimberl, U., Petros, J.A., and Issa, M.M. (2005) Role of transrectal ultrasonography in diagnosis and treatment of retained Foley catheter. *Urology* **65(5),** 1001.
5. Cosman, B.C., Vu, T.T., and Plowman, B.K. (2002) Topical lidocaine does not limit autonomic dysreflexia during anorectal procedures in spinal cord injury: a prospective, double-blind study. *Int. J. Colorectal Dis*. **17(2),** 104–108.
